# People with Easier to Pronounce Names Promote Truthiness of Claims

**DOI:** 10.1371/journal.pone.0088671

**Published:** 2014-02-26

**Authors:** Eryn J. Newman, Mevagh Sanson, Emily K. Miller, Adele Quigley-McBride, Jeffrey L. Foster, Daniel M. Bernstein, Maryanne Garry

**Affiliations:** 1 School of Psychology, Victoria University of Wellington, Wellington, New Zealand; 2 Psychology Department, Kwantlen Polytechnic University, Richmond, British Columbia, Canada; University of Queensland, Australia

## Abstract

When people make judgments about the truth of a claim, related but nonprobative information rapidly leads them to believe the claim–an effect called “truthiness” [1]. Would the pronounceability of others’ names also influence the truthiness of claims attributed to them? We replicated previous work by asking subjects to evaluate people’s names on a positive dimension, and extended that work by asking subjects to rate those names on negative dimensions. Then we addressed a novel theoretical issue by asking subjects to read that same list of names, and judge the truth of claims attributed to them. Across all experiments, easily pronounced names trumped difficult names. Moreover, the effect of pronounceability produced truthiness for claims attributed to those names. Our findings are a new instantiation of truthiness, and extend research on the truth effect as well as persuasion by showing that subjective, tangential properties such as ease of processing can matter when people evaluate information attributed to a source.

## Introduction

In its classic piece, “Clinton Deploys Vowels to Bosnia,” the satirical newspaper The Onion quoted Trszg Grzdnjkln, 44. “I have six children and none of them has a name that is understandable to me or to anyone else. Mr. Clinton, please send my poor, wretched family just one ‘E.’ Please.” The Onion was onto something when it suggested that people with hard to pronounce names suffer while their more pronounceable counterparts benefit.

We know that people think food additives with easier names are safer, amusement park rides less risky, and stocks more lucrative–in fact, the pronounceability of stocks actually translates into real financial gain [2], [3]. Although pronounceability is tangential to decisions about safety, risk, and value, we know that people nonetheless can turn to tangential cues when making judgments.

One such cue is the relative ease of processing information, or its fluency. Fluent (or disfluent) processing can provide metacognitive information about ongoing cognitive operations, and tends to influence judgments most when people have little knowledge or other diagnostic information on which to draw [4], [5], [6], [7]. People tend to interpret fluent processing as a positive cue about a target stimulus, thus evaluating fluent targets along positive dimensions; likewise, they tend to evaluate disfluent targets along negative dimensions (for a review, see [8]; cf. [3]). Pronounceability can give rise to experiences of fluency or disfluency: Accordingly, people say Hnegripitrom is a more hazardous chemical than Magnalroxate; the Tsiischili, a riskier ride than the Ohanzee, and RDO a worse investment than KAR [2], [3].

Even though people’s names carry much information–ethnicity, gender, and socioeconomic status–we are still sensitive to the ease of pronouncing other people’s names when making judgments. In fact, recent work showed that people with easier to pronounce names were evaluated more positively than their harder to pronounce counterparts: They were more likeable, preferred as mock election candidates, and held higher positions in law firms [9]. But the ease of pronouncing a name might have effects that extend beyond the name itself. That is, what we do not know is whether the ease of pronouncing a person’s name might influence not only evaluations about that person, but also information or claims attributed to that person. If we found that information attributed to people with easy to pronounce names is more believable, it would suggest that even basic manipulations of pronounceability can have greater reach than previously thought. Such a finding would not only contribute to the literature on fluency, it would also have implications for many real life instances in which claims are attached to sources with varying ease of pronounceability.

In fact, fluency can have effects that extend beyond a fluent target. That is, fluency can influence judgments about information temporally linked to that target, rather than just the target itself [10], [11]. For instance, in one study people were more likely to choose a bottle of wine when the label featured a picture (such as a frog) that was recently primed–even though the picture had nothing to do with the quality or the name of the wine [11]. In another study, people were more likely to find an argument persuasive when the argument was attributed to a face that subjects had seen earlier in the experiment, even though the familiar face had no diagnostic value for judging the quality of the argument [12]. Taken together, these studies tell us that fluency leaks (see also [13]). But what we do not know is whether the fluency of a name would have effects that extend beyond the name itself, and leak on to judgments of truth–particularly in a single exposure.

We do know that when claims themselves are presented in a way that makes them feel fluent, people rate them as more likely to be true: Claims presented in high colour contrast are rated true more often than claims presented in low colour contrast, and claims that are repeated are rated true more often than claims that are not repeated [7], [14], [15], [16]. We also know, from recent work, that pairing a claim with related but nonprobative information–information that provides no diagnostic information about the accuracy of the associated claim–nonetheless rapidly pushes people to say the claim is true, an effect known as “truthiness” (from comedian Stephen Colbert, who defined truthiness as “truth that comes from the gut, not books.” See also tinyurl.com/truthiness2012) [1], [17], [18]. For example, within seconds, people judge a claim such as “The liquid metal inside a thermometer is magnesium” to be true more often when it appears with a photo of a thermometer than when it appears alone. Likewise, people say claims about celebrities are true if those claims appear with a few words describing a celebrity’s race, sex, profession, and hair than if the claim appears alone.

In those experiments, the data suggest truthiness arises because nonprobative information boosts conceptual processing, helping people generate pseudoevidence that supports the claim. But names are decidedly nonprobative, and should not boost the conceptual processing of accompanying claims, nor help people generate pseudoevidence that those claims are true. If easy to pronounce names rapidly led people to say the accompanying claims were true, it would be a novel route to truthiness.

In Experiments 1a–c we sought to replicate Laham and colleagues’ [9] fluency effects with our materials and extend their findings to negative evaluations (cf. [3]). Thus, in Experiments 1a–c we asked people to evaluate names on either positive or negative dimensions. To control for ethnicity, we created names from various world regions. Within each region, one name was relatively easy to pronounce and the other relatively difficult. Our results suggest that people’s names are like chemicals, roller-coasters and stocks: Across these experiments, easily pronounced names trumped difficult names.

In Experiment 2, we turned to an important and novel theoretical issue, asking to what extent pronounceability of names would produce truthiness for associated claims. We found that even though pronounceability is nonprobative, people with easy to pronounce names–like related photographs and words–confer truthiness on claims.

## Experiments 1a–c

In Experiments 1a–c, we asked whether pronounceability of names influenced how people evaluate unknown others on positive or negative traits.

### Method

#### Subjects

Thirty students at Victoria University of Wellington completed Experiment 1a. In Experiments b and c the corresponding Ns were 28 and 61 (these were convenience samples for a class project). All subjects were recruited from various locations on campus.

#### Ethics Statement

This research was approved by the School of Psychology Human Ethics committee under the delegated authority of the Victoria University of Wellington ethics committee. We obtained written informed consent from all subjects before they participated.

#### Design

We manipulated ease of pronunciation within subjects.

#### Procedure

We used foreign newspapers and websites from 18 countries worldwide, recombining (within each country) real first names with other real last names to create 218 novel combinations of foreign names.

We then created 10 lists, each comprised of a random and unique mix of these names, as well as famous names to provide a benchmark, and asked students in an undergraduate psychology course to “Please rate the ease with which these names of people can be pronounced” on a scale where 1 = very difficult and 7 = very easy. Although the notion of “pronounceability” undoubtedly encompasses a broad range of linguistic features, we did not operationalize it for raters.

We used these rating data to select nine pairs of names. Within each pair, one name was relatively easy to pronounce (mean ratings ranged from 5 to 5.83; e.g., Andrian Babeshko), and the other difficult (mean ratings ranged from 1.17 to 2.67; e.g., Yevgeny Dherzhinsky). Hereafter, we refer to these two categories of names simply as “easy names” and “difficult names.” Then we asked nine raters (other students in the department) to identify from where, geographically, the person from each name hailed to ensure that names in each pair were identified as being from the same region. The same nine raters also reported the number of syllables in each name; easy and difficult names did not differ (M_easy_ = 5.15, SD = 1.39 vs. M_difficult_ = 5.22, SD = .1.40), t(16) <1, orthographic regularity (here, log bigram frequency; M_easy_ = 2.91, SD = 1.02 vs. M_difficult_ = 2.77, SD = .93), t(16) <1 (as calculated by the MCWord Orthographic Wordform database, www.neuro.mcw.edu/mcword). Table 1 shows the final list of 18 names: two pairs from East Asia, South Asia, West Europe, East Europe, and one from the Middle East.

**Table 1:** Names, Classified by Ease of Pronunciation and World Region of Origin.

	World region				
Pronounceability	East Asia	West Europe	Middle East	South Asia	East Europe
Easy	Chen Meina	Bodo Wallmeyer	Amira El-Naggar	Bandula Premachandra	Andrian Babeshko
	Chung Jung-hee	Marciano Larrosa	–	Putali Angami	Lubov Ershova
Difficult	Hur Hye-seong	Maribel Alconero	MahbobehMir-Ma’soum	Shagnik Ravunniarath	Czeslaw Ratynska
	Yu Zhenglong	Svea Gelowicz	–	Shobha Bhattacharya	Yevgeny Dherzhinsky

*Note.* Only two names from the Middle East were presented to subjects.

To avoid a floor effect in Experiment 1a, we asked subjects to read a preamble before rating the familiarity of each name (the 18 names appeared in 10 random orders) on a 5-point scale where 1 = very unfamiliar and 5 = very familiar: “These people are all famous in their respective countries, and you’ve probably heard of them before. But you may not realize you know their names, because you might have an implicit–unaware–memory for their names.” Experiments b and c followed the same method with minor changes, as noted.

In Experiment 1b subjects read, “Imagine you are a tourist looking for a tour guide. You are not feeling very well on the day of your tour and want to avoid the tour leaders who are too risky and adventurous” (cf. [3]). Subjects then saw the names and decided “which tour guides are the most risky (and would make you feel more sick).” They responded on a 5-point scale where 1 = very safe and 5 = very risky.

In Experiment 1c, we asked subjects to “Judge how dangerous the following people are, even if you think you’ve never heard of them before” where 1 = very safe and 5 = very dangerous.

## Results and Discussion

### Experiment 1a

We calculated, for each subject, the mean ratings of familiarity and classified those means by whether the name was easy or difficult (see Datasets S1–S4 for the raw data for each experiment). Subjects rated easy names as more familiar (M_easy_ = 1.55, SD = .69, 95% CI [1.30–1.81]) than difficult names (M_difficult_ = 1.40, SD = .54, 95% CI [1.19–1.60]), t(29) = 2.63, p = .01, Cohen’s d = .53.

### Experiment 1b

The mean ratings of the tour guide’s riskiness reveal a pattern similar to Experiment 1a: subjects rated easy names as less risky (M_easy_ = 2.62, SD = .48, 95% CI [2.43–2.80]) than difficult names (M_difficult_ = 2.86, SD = .43, 95% CI [2.70–3.03]), t(27) = 2.90, p = .01, Cohen’s d = .55.

### Experiment 1c

Even without the positive context of adventure tourism, subjects rated easy names as less dangerous (M_easy_ = 2.46, SD = .60, 95% CI [2.31–2.62]) than difficult names (M_difficult_ = 2.70, SD = .67, 95% CI [2.53–2.87]), t(60) = 4.01, p<.01, Cohen’s d = .52.

Although these findings replicate Laham et al.’s [9] basic findings we still do not know the extent to which effects of pronunciation are limited to the name, or can extend to information attributed to the name. Put another way, would claims attributed to Andrian Babeshko seem truer than those attributed to Yevgeny Dherzhinsky?

## Experiment 2

### Method

#### Subjects

A total of 105 psychology students completed the study for course credit.

#### Ethics statement

This research was approved by the School of Psychology Human Ethics committee under the delegated authority of the Victoria University of Wellington ethics committee. We obtained written informed consent from all subjects before they participated.

#### Design

We used a 2 (trivia statement: true, false) x 2 (name: easy, difficult) within-subject design.

#### Procedure

We told subjects some international students had reported their favorite bits of trivia, some of which were wrong. We paired each trivia claim with a name from Experiments 1a-c, and asked subjects to decide if each claim was true or false. We used Macintosh iBook G4 computers and PsyScope software to present a name for 2 seconds (“Andrian Babeshko said:”); then a claim (“Turtles are deaf”) appeared alongside the name until subjects reported whether it was true or false [19].

Subjects evaluated 16 difficult claims (mean accuracy in norming = 52.34%, SD = 6.27%), half of which were true. To simplify counterbalancing, we used 16 of the original 18 names; half the claims appeared with an easy name and half with a difficult name. We counterbalanced so that each true and false trivia claim appeared equally often with easy and difficult names.

### Results and Discussion

Claims attributed to easy names led to truthiness more than those attributed to difficult names. In other words, a 2 (trivia claim: true, false) x 2 (name: easy, difficult) repeated measures ANOVA showed a main effect for name, F(1,104) = 7.98, p = .01, M_Easy Names_ = .55, SD = .16, 95% CI [.52–.59]; M_Difficult Names = _.50, SD = .18, 95% CI [46–.53]. People also judged true statements as true more often than false statements, performing above chance for true statements and at chance for false statements, F(1,104) = 19.82, p<.01, M_True Statements_ = .58, SD = .19, 95% CI [.55–.62]; M_False Statements_ = .47, SD = .19, 95% CI [43–.50]. There was no interaction, F <1, M_Easy Names True_ = .62, SD = .24; M_Difficult Names True_ = .54, SD = .24; M_Easy Names False_ = .49, SD = .27; M_Difficult Names False_ = .45, SD = .23.

Did the addition of easy names actually make claims seem more trustworthy? Or did the difficult names push people in the direction of disbelieving those claims? Given that the distribution of a confidence interval is a cat’s eye distribution such that the population mean is more likely captured by the center of the CI and increasingly less likely at its ends [20] our data suggest that relative to baseline (52.34%), easy names pushed people toward saying the claims were true, M_Easy Names_ = .55, SD = .16, 95% CI [.52–.59]. These findings suggest that the ease of pronouncing a name can shape distal evaluations of information merely attributed to a name.

## General Discussion

Across four experiments, our findings tell a clear story: People with easy names and their claims are evaluated more favorably relative to their difficult counterparts, for both positive and negative evaluations. Easy names were evaluated as more familiar, less risky and less dangerous (Experiments 1a–1c, respectively). This effect of pronounceability bolsters earlier demonstrations that both things and people with easier to pronounce names are evaluated more positively [2], [3], [9]. Considered together, our findings show that easy names can confer a host of benefits on the people who bear them.

Moreover, Experiment 2 fits with Laham et al. [9], but is not merely a conceptual replication: we discovered a new and interesting route to truthiness [1], [17]. We found that people with easy names confer truthiness on claims relative to people with difficult names. This data pattern demonstrates that the pronounceability of names extends beyond judgments tied to names themselves, and can influence judgments of temporally associated information.

Our findings extend research on the truth effect as well as persuasion [14] (see [21] for a review), [22], [23] (see [24] for a review) by showing that subjective–and tangential–properties such as ease of processing can matter when people evaluate information from a source. Our work also fits with work relating cognitive fluency and ratings of intelligence. In one study, subjects rated simpler essays as more comprehensible (and their authors more intelligent) than complex essays, which suggests that simpler language afforded more fluent processing (Experiments 4 and 5, [25]). It makes sense to evaluate people’s intelligence based on features of their writing (e.g. [26]). But it is surprising that our subjects evaluated the accuracy of other people’s general knowledge or crystallized intelligence [27]–based on phonetic features of their name.

Of course pronunciation ease is just one route to a feeling of easy or difficult processing. For instance simply furrowing one’s eyebrows can make something feel disfluent or difficult to process (e.g. [28], [29]). An interesting question for future research is whether easy or typical names could turn into or act like difficult names under some conditions–perhaps furrowing one’s eyebrows would make “Andrian Babeshko” feel more like “Yevgeny Dherzhinsky”.

Another important avenue for future research is to consider how much information people need about others–and in what circumstances–before they stop relying on fluency to judge them. For example, perhaps people would be less susceptible to the ease of pronunciation if they were evaluating claims attributed not to strangers, but to familiar names such as Brad Pitt. Indeed, the more that people can rely on other diagnostic information–their past experience or general knowledge–to inform their judgments, the less inclined they are to rely on an experience of fluency [6], [7], [30] (see also [31]).

Our results have practical implications. They suggest that when diagnostic information is unavailable, people will turn to pronunciation when evaluating others along a number of dimensions and in a variety of contexts–from assessments of danger through to assessments of truth. Such an effect might have significant real world impact. For instance, would the pronounceability of eyewitnesses’ names shape jury verdicts? This question, too, is an interesting one for future research. Meanwhile, spare a thought for The Onion’s Grg Hmphrs, a resident of Sjlbvdnzv: “With just a few key letters, I could be George Humphries. This is my dream”.

## Supporting Information

Dataset S1 **Familiarity ratings data. Raw data for Experiment 1a.**
(XLSX)

Dataset S2 **Risk ratings data. Raw data for Experiment 1b.**
(XLSX)

Dataset S3 **Danger ratings data. Raw data for Experiment 1c.**
(XLSX)

Dataset S4 **Trivia judgments data. Raw data for Experiment 2.**
(XLSX)
